# Estrogen Receptor-Negative Breast Ductal Carcinoma: Clinicopathological Features and Mib-1 (Ki-67) Proliferative Index Association

**DOI:** 10.1371/journal.pone.0089172

**Published:** 2014-02-28

**Authors:** Noorasmaliza MdPaiman, Siti Aishah Md Ali, Reena MdZin, Meor Zamari Meor Kamal, Wan Anna Md Amin, Mohan Nallusamy, Pavitratha Puspanathan, Rohaizak Muhammad, Sharifa Ezat Wan Puteh, Srijit Das

**Affiliations:** 1 Department of Pathology, Universiti Kebangsaan Malaysia, Kuala Lumpur, Malaysia; 2 Department of Pathology, Sultanah Bahiyah Hospital, Alor Setar, Malaysia; 3 Department of Surgery, Sultanah Bahiyah Hospital, Alor Setar, Malaysia; 4 Department of Surgery, Universiti Kebangsaan Malaysia, Kuala Lumpur, Malaysia; 5 Department of Epidemiology, Universiti Kebangsaan Malaysia, Kuala Lumpur, Malaysia; 6 Department of Anatomy, Universiti Kebangsaan Malaysia, Kuala Lumpur, Malaysia; Health Canada and University of Ottawa, Canada

## Abstract

Breast cancer estrogen receptor (ER) status is one of the strong additional factors in predicting response of patients towards hormonal treatment. The main aim of this study was to assess the morphological characteristics and proliferative activity using MIB-1(Ki-67) of estrogen receptor negative invasive breast ductal carcinoma (NOS type) as well as to correlate these features with clinicopathological data. We also aim to study the expression of c-erbB2 in ER negative breast tumors. High proliferative rate (MIB-1 above 20%) was observed in 63 (63.6%) of 99 ER negative tumors and that these tumors were associated with high expression of c-erbB2 (57.6%). We observed that MIB-1 is a reliable independent prognostic indicator for ER negative infiltrating ductal carcinoma in this study.

## Introduction

Breast cancer is a leading cause of cancer death among women worldwide [1]. In Malaysia, the National Cancer Registry in 2003 had reported 3738 female breast cancer cases and it accounted for 31% of newly diagnosed female cases [2].

Breast cancer estrogen receptor (ER) status is one of the strong additional factors in predicting response of patients towards hormonal treatment, and its determination has become a standard practice in the management of breast carcinomas [3].

Estrogen receptor positive group of tumors appear better differentiated on morphology and bear better prognosis while the clinicopathologic findings of estrogen receptor negative breast carcinomas have been mixed [4]. Despite these inconsistencies, estrogen receptor negative tumors are more chemosensitive than its receptor positive counterpart [3], [5], [6].

Lymph node status and tumor size have long been established as important prognostic factors in predicting disease outcome. However, additional predictive and prognostic factors are required to improve the management of breast cancer as the traditional methods of assessing nodal status and tumor size were found to be insufficiently accurate [7].

Assessment of proliferation rate in breast carcinomas has remained the most important prognostic value [7], [8]. The Ki-67 antigen was the first immunohistochemically detectable marker which recognizes a nuclear epitope present only in proliferating cells. However, formalin fixation causes denaturation changes of the Ki-67 epitope resulting in the development of a monoclonal antibody, MIB-1 which can be easily applied to formalin-fixed paraffin-embedded tissues after heat-mediated antigen retrieval [9]. A pronounced decrease in MIB-1 labeling index has been associated with a good response to preoperative treatment [9], [10], relapse-free and disease specific survival [7], [9]. Higher risk of relapse in both node positive and negative as well as worse survival outcome in early breast cancer has been observed in tumors with MIB-1 positive [7], [11]. Many studies have focused on the utility of Ki-67 in estrogen negative tumors but studies of MIB-1 expression in this group of tumors have been scarce.

c-erbB2 is amplified in approximately 20% of breast cancer [12] and its overexpression is associated with clinical outcomes in patients with breast cancer [13]. Most importantly, studies have shown that c-erbB2 is a useful marker for therapeutic decision making for patients with breast cancer [14].

The main aim of the study was to correlate the morphological features of estrogen receptor negative ductal breast carcinomas with clinicopathological data and other prognostic variables such as stage, grade, axillary lymph node status, age and menopausal status. We also investigated the expression of MIB-1 in estrogen negative breast tumors as well as in triple negative breast tumors and correlate the MIB-1 status in these tumors with clinicopathological data and other prognostic variables.

## Materials and Methods

A retrospective cohort reviewing histological material (blocks and slides) and patient's medical records between January 2003 and December 2007 in Hospital Sultanah Bahiyah, Alor Setar, Kedah, Malaysia was performed. The study was approved by the UKM Medical Centre Ethics Committee (UKM Ethics Committee No: UKM FF-067-2007). The recruitment of samples was based on a universal sampling method whereby all patients diagnosed with primary breast invasive ductal carcinoma (Non Otherwise Specified - NOS) with immunohistochemically confirmed estrogen receptor (ER) negative were taken for the study. Determination of the ER-negative breast cancer were done by the reporting pathologists and were recorded in the histopathological reports.

A total of 477 breast cancer cases (NOS and special types) were identified with 138 found to be ER-negative. However, only ninety-nine cases were included in this study based on the availability of the tissue blocks in the laboratory. The clinicopathological data (clinical staging, tumor grading, lymph node status, menopausal status, progesterone receptor and c-erbB2 status) was obtained from the medical records, and the morphological features were reviewed from the representative hematoxylin and eosin-stained available slides by two independent pathologists.

### Immunohistochemical staining method for MIB-1

Monoclonal mouse anti-human Ki-67 antigen (Clone MIB-1; DAKO, USA; dilution 1∶150) and a representative tissue block was prepared for MIB-1 immunohistochemical stain according to the manufacturer's instructions.

Sections of 2.5–3μ were cut from the selected paraffin blocks and applied on poly-L-lysine coated slides. Slides were taken to water through three changes of xylene followed by rehydration through graded alcohol prior to subjecting the slides to antigen retrieval using the pressure cooker method. The slides were then incubated in 3% hydrogen peroxide 3% for 5 minutes and later washed with distilled water, followed by Tris buffered saline (TBS). Following pretreatment, the Ki-67 and ER antibodies were applied to the slides and incubated for 30 minutes each at dilution of 1∶150 and 1∶100 respectively. After washing with TBS twice, sections were incubated with the polymer for 30 minutes and again rinse twice in TBS. Dako liquid DAB substrate (Dako REAL™EnVision™ Detection system) was used as a chromogen and sections were counterstained with hematoxylin. Positive controls were stained with the primary antibody. On the other hand, the primary antibody was omitted in negative controls.

### Evaluation of clinicopathologic features of ER negative breast cancer

The demographic findings, clinical outcome and tumor characteristics of patients with ER negative tumors were analysed along with the morphological features (tumor margin, stromal inflammation, comedo-type necrosis and tumor giant cells).

### Evaluation of Immunohistochemical Staining

MIB-1 and ER immunohistochemical status in breast cancer were evaluated by reporting pathologists, double blinded to the clinicopathological data.

### MIB-1

**Figure 1:**
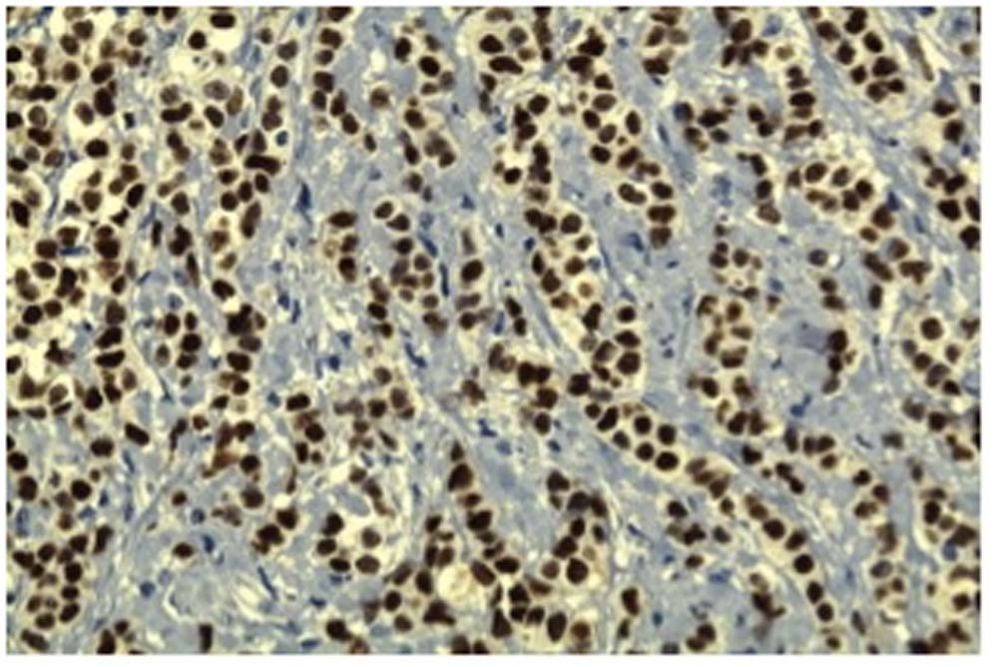
Distinct nuclear immunoreactivity for MIB-1 positive (>20%) in ER negative breast cancer (×100 magnification).

Malignant cells with distinct nuclear staining were interpreted as positive. For interpretation, MIB-1 index is expressed semi quantitatively only in the invasive component of the tumor. A cut - off point of ≥20% positive cells (Figure 1) were selected to define “positive” (i.e. high risk) cases based on the findings of previous studies [10]. Malignant cells with faint nuclear staining as well as quantitatively less than 20% positive of the tumor were considered negative. Formalin fixed tonsillar tissue was used as the positive control. Formalin fixed breast cancer tissue with omitted primary antibody was used for negative control. All the controls were included in every batch to ensure validity of the staining.

### ER

**Figure 2:**
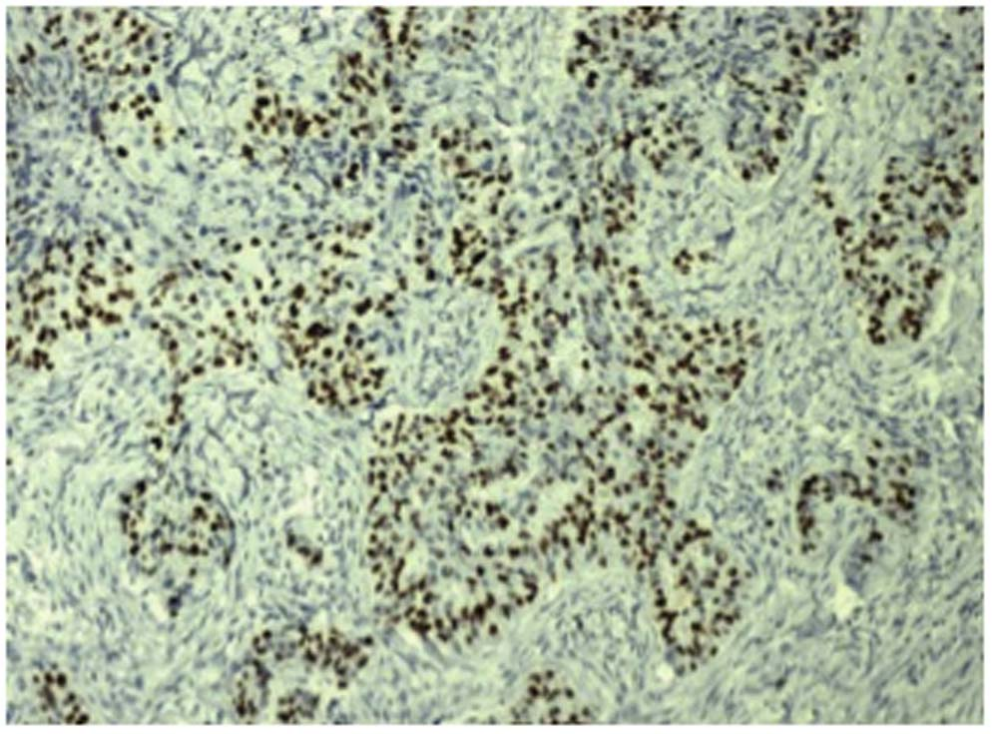
Immunohistochemical stain for ER in invasive breast carcinoma showing strong nuclear positivity (×200 magnification).

**Figure 3:**
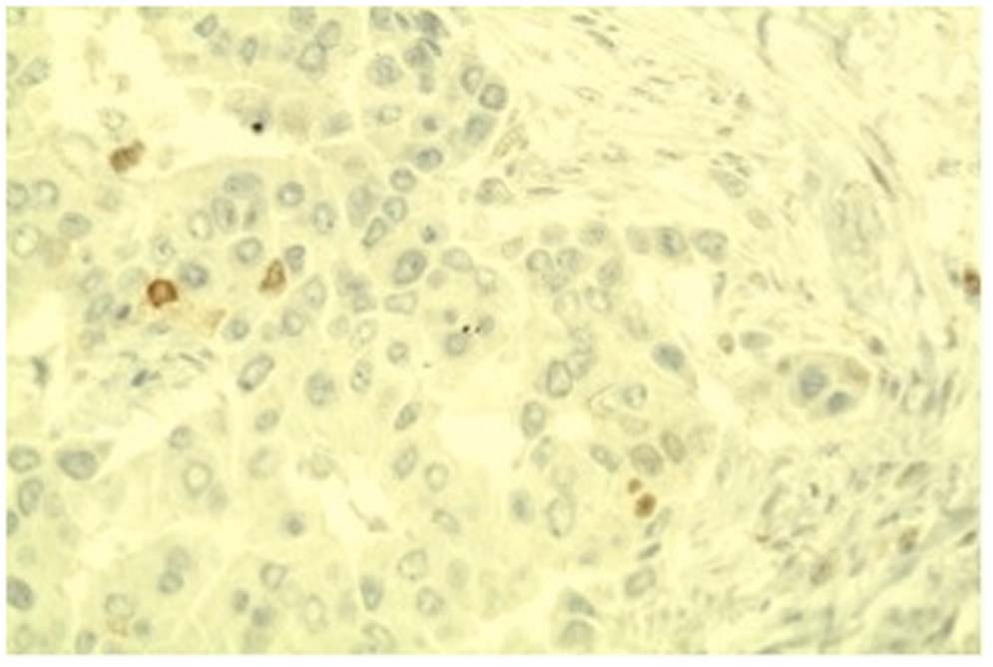
Immunohistochemical stain showing negative ER immunostaining in invasive breast carcinoma (×200 magnification).

ER status determination in breast cancers was performed by reporting pathologists. ER immunostaining was evaluated in the nuclei of malignant cells and scored as either positive or negative. A 10% cut-off threshold value of the entire tumor cell nuclei population was selected, based on previous studies [15]–[17]. Breast cancers with positive and negative ER immunostaining were shown (Figures 2 and 3).

### Statistical analysis

Data was statistically analysed with SPSS version 14.0 statistic software. Association of ER negative breast carcinoma with morphological features and proliferative activity, as well as clinicopathological data were carried out by Pearson's Chi-Square test analysis. A p-value<0.05 was considered significant.

## Results

### Demographic findings, clinical outcome and tumor characteristics of patients with ER negative tumors

The demographic findings, clinical outcome and tumor characteristics of patients with ER negative tumors were tabulated (Table 1).

**Table 1:** Clinical outcome of ER-negative tumor cases (within 0 to 5 years follow up) in relation with morphological and clinicopathological data.

	Clinical outcome (0 to 5 years follow up)						
	Survive and healthy	Survive with local recurrence	Survive with metastasis	Died	Defaulted	Kulim/unknown	Referred to other hospital
	n (%)	n(%)	n(%)	n(%)	n(%)	n(%)	n(%)
**Total number of patients, 99**	38(38.4)	2 (2)	2 (2)	12 (12.1)	9 (9.1)	33 (33.3)	3 (3)
**Age (years)**							
20–30	0	2	0	0	0	1	0
31–40	6	0	0	1	0	10	1
41–50	13 (34.2)	0	1	4 (33.3)	0	11	1
51–60	12 (31.6)	0	0	1	4	9	1
≥61	7	0	1	6 (60)	5	2	0
**Menopause**							
Pre	18	2	1	5	1	24	2
Post	20	0	1	7	8	9	1
**Tumor size**							
≤2 cm	10	1	0	0	1	2	0
>2 cm	28	1(50)	2(100)	12(100)	8	31	3
**Lymph node status**							
Positive	27	1(50)	2(100)	11(91.7)	4	28	2
Negative	10	1	0	0	3	3	1
Not known	1	0	0	1	2	2	0
**Tumor grade**							
1	2	0	0	0	1	0	0
2	11	1	0	1	1	5	1
3	25	1(50)	2(100)	11(91.7)	7	28	2
**Tumor staging**							
I	5	1	0	0	0	1	0
II	18	0	0	1	6	11	1
III	15	1	2	8	3	21	2
IV	0	0	0	3	0	0	0
**Comedo necrosis**							
Present	22	2(100)	1(50)	8(66.7)	5	16	2
Absent	16	0	1	4	4	17	1
**Tumor giant cell**							
Present	26	2(100)	2(100)	11(91.7)	7	23	3
Absent	12	0	0	1	2	10	0
**Tumor margin**							
Pushing	13	0	1	2	4	13	1
Infiltrative	25	2(100)	1(50)	10(83.3)	5	20	2
**Stromal inflammation**							
Present	16	0	1	2	4	8	2
Absent	22	2(100)	1(50)	10(83.3)	5	25	1
**PR status**				)			
Positive	9	0	0	1	0	5	0
Negative	29(76.3)	2(100)	2(100)	11(91.7)	9(100)	28(84.8)	3(100)
**c-erbB-2 status**							
Positive	23(60.5)	2	1(50)	7(58.3)	7(77.8)	18	1
Negative	15	0	1	5	2	15	2
**MIB-1**							
Positive (≥20%)	27(71)	2(100)	2(100)	6(100)	5	19	2
Negative(<20%)	9	0	0	0	4	12	1
Block not available	2	0	0	0	0	2	0

**Figure 4:**
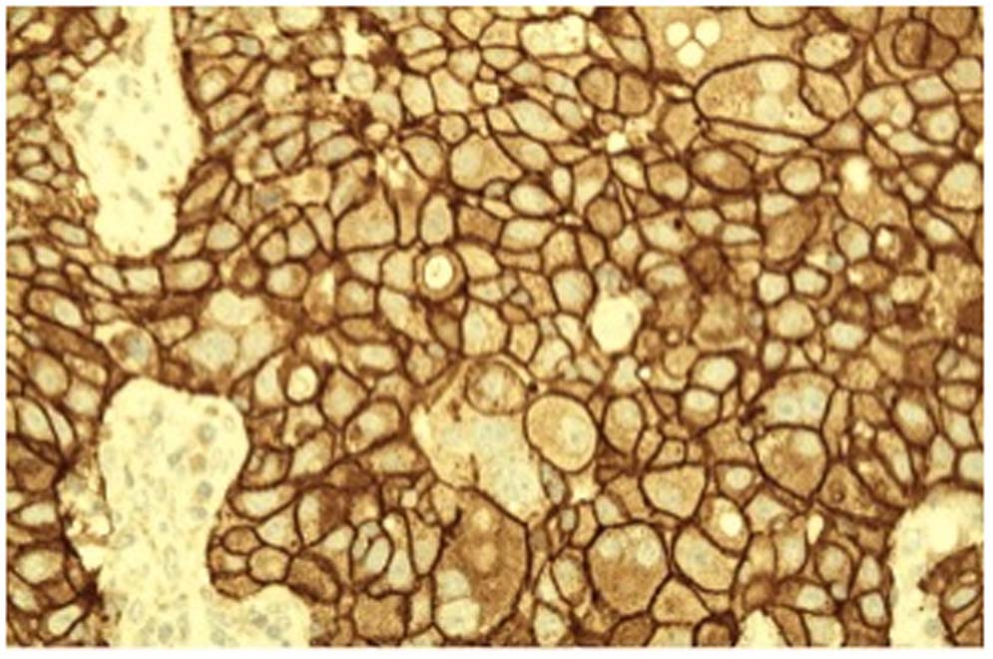
C-erbB2 overexpression shows strong positivity (3+) on the cell membrane by immunohistochemistry (×200 magnification).

All the 99 patients were female with an age range from 20 to 70 years with a peak at 41–50 years. Among known menopausal status, 53.5% (53/99 cases) and 46.5% (46/99 cases) of patients were premenopause and postmenopause, respectively. Most of the tumors are ≥2 cm in size (85/99 cases; 85.9%), in which 23 out of 85 cases were 2 cm to 5 cm in size. There were 75/99 cases (75.8%) with lymph node metastases (positive), while 37/99 cases (37.4%) were stage II and 52/99 cases (52.5%) were stage III. In comparison, only 18.2% of the patient (18/99 cases) in the entire series of ER-negative tumors had no lymph node metastases (negative). A high proportion of tumor was graded 3 (76/99 cases; 76.8%) followed by grade 2 (20/99 cases; 20.2%) and only 3 cases of grade 1 (3%). A total of 84.4% of cases (84/99) showed PR negative, while 57.6% of cases (57/99) was c-erbB2 positive (Figure 4).

The presence of comedo-type tumor necrosis (56/99 cases; 56.6%) tumor giant cells (74/99 cases; 74.7%) and infiltrative margin (65/99 cases; 65.6%) as well as absence of stromal inflammation (66/99 cases; 66.7%) were the most common morphological features seen in these tumors.

### Association between grade, stage and morphologic features in ER negative breast tumors

In ER negative breast tumors, there was strong association between tumor grade 3 with stage III [p = 0.014], with tumor size more than 2 cm [p<0.000], and axillary lymph node metastases [p = 0.05] (Table 2). Tumor grade 3 was also more likely to be seen in ER negative breast tumors of postmenopausal patient [p = 0.040, data not shown]. When the tumor morphological features were compared to lymph node status, the presence of tumor infiltrative margin showed significant relationship with axillary lymph node metastases [p = 0.016] in ER negative breast tumor (Table 3).

**Table 2:** Significant correlation between tumor staging and tumor size, tumor grade, lymph node metastases in ER-negative tumor.

	Stage I	Stage II	Stage III	Stage IV	p-value
Tumor size					
≤2 cm	6	8	0	0	<0.000
>2 cm	1	29	52	3	
Tumor grade					
1	0	3	0	0	0.014
2	4	10	6	0	
3	3	24	46	3	
Lymph node metastases					
Positive	1	19	52	3	<0.000
Negative	5	13	0	0	
Unknown	1	5	0	0	

Correlation is significant at the 0.05 level (2-sided).

**Table 3:** Significant correlation between axillary lymph node metastases and tumor margin in ER-negative tumor.

	Lymph node metastases			p-value
	Positive	Negative	Unknown	
Tumor margin				0.016
Pushing	20	11	3	
Infiltrative	55	7	3	

Correlation is significant at the 0.05 level (2-sided).

### Association between MIB-1 and morphologic features and clinicopathologic data of ER negative breast tumors

MIB-1 was positive in 63 (63.6%) of 99 ER negative breast tumors (Table 4), however, this was found not to be significant. By Pearson's Chi Square test analysis, there was significant inverse association between expression of MIB-1 and stromal inflammation [p = 0.05]. There was no significant association between MIB-1 and other morphologic features (p>0.10) as well as clinicopathological data (p>0.8) [data not shown].

**Table 4:** Correlation between MIB-1 status with clinicopathological findings and morphologic features.

Variables	Number of cases, n(%)	MIB-1 positive, n (%)	MIB-1 negative n(%)	MIB-1 unknown n(%)	p-value
**Total number of patients**	99(100)	63(63.6)	33(33.3)	4(4)	
**Age (years)** 20–30	3 (3.0%)	3	0	0	0.58
31–40	18 (18.2%)	10	8 (8.1)	0	
41–50	30 (30.3%)	21(21.2%)	9(9)	0	
51–60	27 (27.3%)	16	7(7)	4(4)	
≥61	21 (21.2%)	13	8 (8.1)	0	
**Menopausal status** Pre	53 (53.5%)	36(36.4%)	16 (16.1)	1(1)	0.45
Post	46 (46.5%)	27	16 (16.1)	3(3)	
**Clinicopathological data**					
**Tumor size** ≤2 cm	14 (14.1%)	9	5(5.1)	0	0.89
>2 cm	85 (85.9%)	54(54.5%)	27 (27.3)	4(4)	
**Axillary lymph node** Positive	75 (75.8%)	46(46.5%)	26 (26.3)	3(3)	0.29
Negative	18 (18.2%)	14	3(3)	1(1)	
**Tumor grade** 1	3 (3.0%)	2	1(1)	0	0.39
2	20 (20.2%)	11	9(9)	0	
3	76 (76.8%)	50(50.5%)	22 (22.2)	4(4)	
**PR** Positive	15 (15.2%)	13	2(2)	0	0.05*
Negative	84 (84.8%)	50	30(30.3)	4(4)	
**c-erbB-2** Positive	57 (57.6%)	32	22(22.2)	3(3)	0.11
Negative	42 (42.4%)	31	10(10.1)	1(1)	
**Tumor stage** I	7 (7.1%)	6	1(1)	0	0.63
II	37 (37.4%)	22	13(13.1)	2(2)	
III	52 (52.5%)	34(34.3%)	16(16.1)	2(2)	
IV	3 (3.0%)	1	2(2)	0	
**Morphological features**					
**Comedo-type necrosis** Present	56 (56.6%)	36(36.4%)	17(17.2)	3(3)	
Absent	43 (43.4%)	27	15(15.2)	1(1)	
**Tumor giant cells** Present	74 (74.7%0	46(46.5%)	25(25.3)	3(3)	
Absent	25 (25.3%)	17	7(7)	1(1)	
**Tumormargin** Infiltrative	65 (65.7%)	42(42.2%)	22(22.2)	3(3)	0.92
Pushing	34 (34.3%)	21	10(10.1)	1(1)	
**Stromal inflammation** Present	33 (33.3%)	24	6(6)	3(3)	0.05*
Absent	66 (66.7%)	39(39.4%)	26(26.3)	0	

Correlation is significant at the 0.05 level (2-sided).

### MIB-1 protein in triple negative tumors (ER, PR and c-erbB2 negative) and the association with clinicopathologic features

A total of 99 cases of ER negative breast cancer was identified out of which 36 of these showed concurrent lack of immunoreactivity in both PR and c-erbB2 (36%, Table 5). These cases were thus classified as triple negative breast tumors. MIB-1 protein was expressed in 69% of triple negative tumor cases (25/36).

**Table 5:** Frequency table of clinicopathological data and morphologic features of triple negative breast tumor.

Variables	Number of cases (%)
	(Total patient – 36)
**Age (years)** 20–30	3 (8.3%)
31–40	7 (19.4%)
41–50	10 (27%)
51–60	8 (22%)
≥61	8 (22%)
**Menopausal status**	
Pre	20 (55%)
Post	16 (44%)
**Morphological features**	
**Tumor margin** Pushing	13 (36.1%)
Infiltrative	23 (63.9%)
**Stromal inflammation** Present	8 (22%)
Absent	28 (77.8%)
**Comedo-type necrosis** Present	23 (63.9%)
Absent	13 (36.1%)
**Tumor giant cell** Present	30 (83.3%)
Absent	6 (16.7%)
**Clinicopathological data**	
**Tumor size** ≤2 cm	4 (11.1%)
>2 cm	32 (88.9%)
**Tumor grade** 1	2 (5.6%)
2	5 (13.9%)
3	29 (80.6%)
**Lymph node status**	
Positive	26 (72.2%)
Negative	9 (25%)
Unknown (wide excision)	1 (2.8%)
**MIB-1** Positive	25 (69.4%)
Negative	10 (27.8%)
Not available	1 (2.8%)
**Tumor stage** I	3 (8.3%)
II	8 (22%)
III	25 (69.4%)
IV	0 (0%)

A total of 90% (32/36) of the triple breast negative tumor cases with MIB-1 immunoreactivity showed tumor size of more than 2 cm while lymph node positivity was involved in 72% of cases (26/36). Approximately 80% of the triple negative breast tumors were grade 3 (29/36 cases) and 69% were stage III (25/36 cases).

Similar to those seen in ER negative (non triple negative) breast tumors, there was also strong association between grade 3, MIB-1 immunoreactive, triple negative breast tumors with tumor size of more than 2 cm [p = 0.001] and lymph node metastases [p = 0.024]. Stage III, MIB-1 immunoreactive, triple negative breast tumors were also strongly correlated with tumor size of more than 2 cm [p<0.000], axillary lymph node metastases [p<0.000] and tumor grade 3 [p<0.000; data not shown]. These findings were summarized in Table 6.

**Table 6:** Correlation between tumor grade and menopausal status, tumor size, lymph node metastases in MIB-1 triple negative breast cancers.

	Tumor grade			p	Tumor Stage				p
	1	2	3		I	II	III	IV	
Tumor size									
≤2 cm	0	3	1	0.001	3	1	0	0	<0.000
>2 cm	2	2	28		0	7	25	0	
Lymph node metastases									
Positive	0	2	24	0.024	0	2	24	0	<0.000
Negative	2	3	4		3	5	1	0	
Unknown	0	0	1		0	1	0	0	

Correlation is significant at the 0.05 level (2-sided).

Morphologically, MIB-1 immunoreactive triple negative breast tumors display comedo-type necrosis and infiltrative tumor margin each in 64% (23/36 cases), tumor giant cells in 83% (30/36 cases) and lack of stromal inflammation in 78% (28/36 cases). However, there was no significant association seen between MIB-1 triple negative tumors with any of these morphologic features.

## Discussion

The role of hormone receptors as a prognostic and therapeutic tool is widely accepted, and estrogen receptor has proven to be a successful target for all ER-positive breast carcinomas [4]. In order to reduce breast cancer mortality, there is a need to further examine and characterize ER-negative tumors, which are traditionally of poor prognosis and lack effective chemopreventive strategies [4].

In the present study, a majority of ER-negative tumors was of grade 3 (76.8%), had axillary lymph node metastases (75.8%) and are also MIB-1 positive (63.6%). These results are similar to previous reports indicating that ER-negative tumor status statistically correlated to histologic grade 3, axillary lymph node metastases and MIB-1 positive [18]. More than 50% of ER-negative tumor in this study showed comedo-type necrosis, which was reported to be characteristic of early development of systemic metastases with an accelerated clinical course [19]. Confluent tumor necrosis of any dimension was reported to be an independent predictor for early recurrence and death from disease [20].

In this study, infiltrative margin showed significant association with axillary lymph node metastases (p = 0.016). This finding was in accordance with an earlier report that ER-negative cancers with pushing margin showed significant correlation with negative lymph node status, suggesting its aggressive behavior [4].

The findings of the present study also showed strong association between ER-negative tumors and tumor grade 3 with tumor size greater than 2 cm. This was consistent with previous studies, which reported association between increasing tumor grade and increased size with ER-negativity [4], [18].

In the present study, MIB-1 positivity showed significant association with PR negative status and absence of stromal inflammation, but not with other clinicopathologic and morphologic features. This was contradictory to previous finding, which showed statistical association between high MIB-1 scores and increasing tumor size, young age and high-grade tumor [9]. Despite the lack of association between MIB status and other clinicopathologic and morphologic features in this study, MIB-1 positive status indicates increased proliferation rate and tumor potential growth in tumors with ER, PR negative status, supportive of other studies [9], [21]. Patients with ER negative tumors are associated with shorter disease - free survival [22] and that stromal inflammations are thought to be impaired in advanced stages of breast cancer [23]. In ER positive, low-grade breast cancers, increased proliferation rate of stromal cells associated with inflammation were shown to have a higher recurrence rate [24]. Although no such observation has yet been found in ER negative tumours, the results of this study suggest cross talk between inflammatory cells and highly proliferative ER negative breast carcinomas.

Ki-67/MIB-1 is useful as a marker of a good chance of response to medical therapy and also been found to be associated with a higher risk of relapse [7]. An earlier study showed statistical correlation between elevated Ki-67 status and high histological grade [18]. In this study, almost 50% (50/99 cases) of grade 3 ER-negative tumors were MIB-1 positive. The prognostic outcome of patients with tumors displaying high proliferative activity is also worse [25]. This was shown in the present study that all patients who died and experienced distant metastases, and local recurrence (2/2 cases) were MIB-1 positive.

### Conclusion

In summary, ER-negative breast cancers are a distinct group of tumors with several unique morphological features. High grade, infiltrative margin, lack of lymphoid stroma, comedo-type necrosis and tumor giant cells are dominant morphological findings. These ER- negative lesions are also predominantly grade 3 carcinomas, a finding that correlates with the absence of stromal inflammation and tumor size greater than 2 cm.

We also observed that MIB-1 significantly correlated with PR hormonal status and stromal inflammation in ER negative breast cancers. MIB-1 was also found to be positive in more than 50% of ER negative tumours. Hence, ER/PR negative breast cancers are therefore tumors of high proliferating index. Given that tumors with high proliferative index occurs in patients with poor clinical outcome, MIB-1 is a potentially reliable prognostic marker in this hormonally resistant subtype of breast cancers.
